# Recombinant FSH Improves Sperm DNA Damage in Male Infertility: A Phase II Clinical Trial

**DOI:** 10.3389/fendo.2018.00383

**Published:** 2018-07-10

**Authors:** Nicola Colacurci, Vincenzo De Leo, Giovanni Ruvolo, Paola Piomboni, Francesca Caprio, Rosario Pivonello, Enrico Papaleo, Eugenio La Verde, Raffaella Depalo, Monica Lispi, Salvatore Longobardi, Donatella Paoli, Francesco Pallotti, Francesco Lombardo

**Affiliations:** ^1^Department of Gynecological, Obstetrics and Reproduction Sciences, II University of Naples, Naples, Italy; ^2^Department of Molecular and Developmental Medicine, Centre for Couple Sterility, University of Siena, Siena, Italy; ^3^Obstetrics and Gynecology, Casa di Cura Candela, Palermo, Italy; ^4^Department of Endocrinology, University of Naples “Federico II”, Naples, Italy; ^5^Operating Unit of Gynecology and Obstetrics, DIMER Center of Natality Sciences, IRCCS San Raffaele of Milan, Milan, Italy; ^6^Pathophysiology Center of Human Reproduction and Freezing Gametes, Ospedale Policlinico Consorziale of Bari, Bari, Italy; ^7^Merck Serono S.p.A, Rome, Italy; ^8^Laboratory of Seminology- Sperm Bank “Loredana Gandini”, Department of Experimental Medicine, Sapienza University of Rome, Rome, Italy

**Keywords:** male infertility, sperm DNA fragmentation, DNA fragmentation index, functional hypogonadotropic hypogonadism, recombinant FSH

## Abstract

**Background and objectives:** Male infertility is a global health dilemma and Follicle-Stimulating Hormone (FSH) administration has shown promising results. Several studies showed that infertile men with normal semen parameters have low levels of DNA damage while infertile men with abnormal semen parameters have more damage at the DNA level. Sperm DNA damage may affect the reproductive outcome and has been associated with failure in the achievement of competent embryos and pregnancy fulfillment. The aim of this study was to evaluate whether the administration of recombinant FSH (Gonal-f® PEN 900 IU) could improve sperm DNA fragmentation in men with infertility. The secondary endpoints of this study were to evaluate the FSH effects on sperm parameters and hormonal assets.

**Methods:** A longitudinal, prospective, multicenter, open-label clinical trial was carried out. Infertile couples were recruited from six Italian Reproductive Medical Centers and 115 infertile men were enrolled for this study. All participants were treated with subcutaneous injections of Gonal-f® 150 IU every other day, within a 3 month-time frame. The semen samples were examined in accordance to the 2010 World Health Organization criteria. Sperm DNA Fragmentation (DFI) was determined by fluorescence microscopy using terminal deoxynucleotidyl transferase-mediated d-UTP Nick-end Labeling (TUNEL) assay. Statistical analysis was performed using both the *t*-test for paired samples and the Wilcoxon signed-rank test.

**Results:** FSH administration improved DFI in 67% of patients, with an average decrease of 35.4% compared to the baseline. This improvement is more evident in men with basal DFI lower than 17% and in those with FSH basal levels between 2.16 and 4.27 IU/L. In addition, FSH enhanced the gonadal function, increasing the hormones AMH and Inhibin B and semen parameters. Limitation of these results are represented by the absence of a placebo group and of FSHR genotype stratification sub-analysis.

**Conclusion:** Recombinant FSH 150 IU is well tolerated and effective in eliciting a significant DFI reduction as well as in improving gonadal function.

Trial Registration: EUDRACT Number 2010-020196-23. Registred 14 April 2011.

## Introduction

Male unexplained infertility defines those cases in which men are infertile despite having normal semen analysis, normal history and physical examination and when female factor infertility has been ruled out (1). Male infertility, a current global health dilemma, affects approximately 10–15% of male adults and up to one in six couples, and in 30–40% of cases, a definite cause for infertility cannot be found (2, 3). In this context, abnormalities in the sperm DNA integrity should be researched and several studies showed that infertile men with normal semen parameters have low levels of DNA damage while, infertile men with abnormal semen parameters have more damage at the DNA level (4). Moreover, up to 8% of infertile men showed an altered DNA integrity together with normal semen parameters (5).

The role of Follicle-Stimulating Hormone (FSH) on spermatogenesis is widely demonstrated both in animals (6) and in humans (7, 8). As a confirmation of this, the use of FSH 150 IU in combination with human Chorionic Gonadotropin (hCG) 3 times a week for up to 18 months, is effective at initiating spermatogenesis in the vast majority of cases of azoospermia due to Hypogonadotropic Hypogonadism (HH) (9). Starting from this point, the administration of FSH has been proposed in cases of oligoasthenoteratozoospermia with low-normal FSH serum levels, using the same FSH doses proposed in the HH. These cases represent a functional HH and the hormonal pattern of these patients suggests that gonadotropins are not able to maintain normal spermatogenesis, although they are in the low-normal range (9). Thus, these infertile patients could benefit from a gonadotropin-based treatment. While in other countries FSH is not approved for male infertility treatment, in Italy, the note number 74 of the Italian Medicines Agency (AIFA) approves the use of FSH in infertile men who want to conceive with “Hypogonadotropic Hypogonadism and normal or low gonadotropin levels, with FSH < 8 IU/L” (10). Several clinical trials, evaluated the effectiveness of the recombinant FSH treatment in improving the sperm quality in male subjects with idiopathic infertility, although no clear, distinct results have been obtained as of yet (11). A recent meta-analysis confirmed that an average of 12 week of FSH treatment improves the pregnancy rate, both in natural cycles and after assisted reproductive techniques, independently from the FSH preparation used (recombinant/purified), but without any clear correlation with duration of FSH administration or cumulative dose. Also, the Authors stated that FSH administration showed significant benefits on sperm concentration, but not on sperm progressive motility (11). Moreover, Simoni et al. recently confirmed the efficacy of FSH in men with idiopathic infertility, performing a multicenter, longitudinal, prospective, open-label, two-arm clinical trial in which the sperm DNA Fragmentation Index (DFI) was used as a primary endpoint (12). However, adequately driven clinical trials are still needed to strengthen the knowledge of the FSH efficacy in this setting.

Semen analysis remains the main tool used in the clinical practice to evaluate male fertility. The sperm parameters evaluated are: volume, sperm concentration, total sperm number, motility and morphology (13). However, seminal parameter values, concerning fertile and infertile men, have a wide overlap, and increasingly stringent parameters should be used to evaluate the male reproductive capability before and after a pharmacological therapy (14). DFI has recently drawn the attention in the research field of human reproduction. Although there is evidence of successful Intracytoplasmic Sperm Injection (ICSI) cycles, it has been shown that such parameters might negatively affect the reproductive outcome, both in natural cycles and in assisted reproduction settings (14, 15). Sperm DNA damage has been associated with failures in the achievement of competent embryos and pregnancy fulfillment (15), and the DFI represents the best tool to evaluate sperm DNA damage (16).

The aim of this clinical study (EUDRACT Number 2010-020196-23) was to evaluate the effects of the recombinant FSH administration, 150 IU every other day in a 3-month time frame, on sperm DNA damage evaluated through DFI in male partners of infertile couples with low-normal FSH levels. The secondary endpoints of this study were to evaluate the FSH effects on sperm parameters and hormonal assets.

## Materials and methods

### Study design

**Figure 1:**
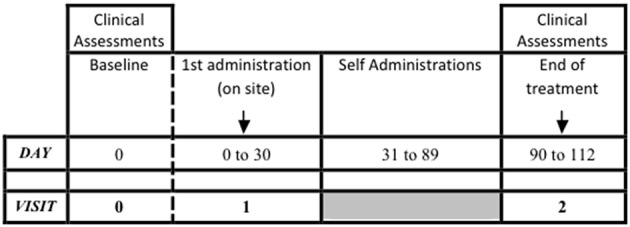
Study design.

A longitudinal, prospective, interventional, open-label, clinical trial was carried out. Three visits were carried out, performed at baseline (Visit 0), at 1 month (Visit 1) and at 4 months (Visit 2) from baseline. During Visit 0, infertile couples were evaluated and the male partner was checked for inclusion and exclusion criteria. Visit 1 coincided with the FSH treatment start, and it was performed within 30 days from the baseline, in accordance to the clinician's judgement. The treatment phase lasted 3 months and ended at Visit 2. The maximum study duration per patient was 4 months. The study design is summarized in Figure 1.

During the baseline visit, the Endocrinologist at each Center evaluated the patients concerning the anamnesis, the physical examination and the testicular ultrasound. Semen and blood samples were obtained at Visits 0 and 2 to evaluate the pre- and post-treatment effects of the FSH administration (at Visits 0 and 2, respectively). The semen and the blood samples were sent to the Central Laboratory within 3 days after being collected.

### Patients

The protocols and the patient informed consents were reviewed and approved by the local Ethics Committees (ECs) of the involved recruiting sites. The Ethics Committee of the II University of Naples released the EC approval of the National Coordinating Site. The study origin and conduct were in accordance with the Declaration of Helsinki as amended by the 59th WMA General Assembly, Seoul, Republic of Korea, October 2008, and compliant with the GCP guidelines and relevant Italian regulation.

Recruitment took place at six Italian Reproductive Medicine Centers in which the male partner of the selected infertile couple was thoroughly evaluated (Rome, Naples, Siena, Palermo, Milan, Bari), through a competitive recruitment model. The first patient was enrolled on July 8th 2011. The last study visit was performed on February 28th 2013. Patient informed consent for the pre-screening procedures was obtained prior to the eligibility assessment.

In the enrolment phase, the infertile couples (no pregnancies after at least 12 months of unprotected intercourse) in which the male partner presented the following inclusion criteria, were considered: (i) age between 18 and 45 years, (ii) total sperm number of >10 million/ejaculate, (iii) total sperm motility of >5% and ≤25%, and (iv) FSH value at baseline >1 and <8 IU/L. Although not strictly related to the male infertility definition (13), these parameters were chosen, taking into consideration the FSH rules provided by the AIFA note number 74 of the FSH together with the minimum number of sperm required for the chromatin integrity analysis. Thus, patients with both unexplained infertility and idiopathic infertility were included. Moreover, during the first visit, obvious female infertility causes have been excluded through the anamnesis.

Each patient was evaluated at the enrolling Center and the following conditions were evaluated in accordance to their clinical practice as exclusion criteria: HH, history of testis trauma, torsion, or prior vasectomy reversal, documented presence of urogenital tract infections, any clinically important systemic disease, any diagnosed or suspected malignant androgen dependent tumors, genetic disorders; documented presence of varicocele by physical examination and confirmed by ultrasound (III degree or higher at the ultrasound evaluation) (17), documented presence of hydrocele, and no medical or surgical treatment in the 3 months prior to the study. Moreover, patients with known genetic causes of male infertility (karyotype abnormalities, and *CFTR* gene mutations, etc.) have been excluded. Smoking behavior represented a criterion for exclusion only when a current attitude to 20 or more cigarettes/day was discovered or a former attitude of 41 or more cigarettes/day with discontinuation within 6 months before the screening visit. Considering exclusion criteria, smokers were included in the study, only when the attitude was less than 20 cigarettes per day.

### Treatment

All enrolled patients received the recombinant FSH (Gonal-f® Pen) treatment, subcutaneously administered, at a 150 IU dosage every other day, starting from Visit 1, for a total period of 3 months. An authorized and trained member of the research staff administered the first injection at the designated research center. The subsequent administrations were performed in other medical facilities or at home, as found suitable for each case. In case of self-administration, the research center instructed the patient on the injection protocol to be carried out. The investigator advised the patient on the injection sites to be chosen. The site staff ensured that the self-administering patients properly followed the protocol.

The Sponsor of the trial, Merck Serono, supplied study medication for this protocol. The Gonal-f® Pen 900 IU is a disposable, pre-filled drug delivery system intended for subcutaneous injection of multiple and variable doses of liquid formulation of follitropin alfa. The product was to be stored in a refrigerator (2–8°C), never under frozen conditions, and protected from direct light source. Before opening and within its shelf-life, the medicinal product could be removed from the refrigerator, without being refrigerated again, for up to 3 months at/or below 25°C temperatures. The product had to be discarded if it had not been used after 3 months. Once opened, the medicinal product could be stored for a maximum of 28 days at/or below 25°C temperatures. The investigator had to write on the Gonal-f pre-filled pen, the day of its first use. Each study kit, which was dispensed to the participants, was made up of 8 (eight) Gonal-f® pre-filled devices.

### Laboratory analysis

Blood analyses were performed at the Core Laboratory of Seminology (Department of Experimental Medicine University of Rome “La Sapienza,” Italy). The hormone parameters were evaluated in a peripheral blood sample collected in the morning (7:30–9:00 a.m.), after fasting overnight. The samples were centrifuged after 30' and the serum was immediately frozen at −20°C temperature. All tests were performed in duplicate. FSH, Luteinizing Hormone (LH), testosterone, and Sex Hormone Binding Globulin (SHBG) were measured by Chemiluminescent Micro-particle Immunoassay (CMIA, Architect System) (Abbott Laboratories, IL, USA), with limits of detection set at 0.05 IU/L, 0.07 IU/L, 0.0144 ng/ml and 0.1 nmol/L, respectively. The normal adult ranges for our laboratory are FSH 1.38–9.58 IU/L, LH 1.80–8.16 IU/L, testosterone 2.8–11.0 ng/ml, SHBG 11.7–78.1 nmol/L. The Inhibin B serum and the AMH were measured by an enzymatic immunoassay (ELISA, Pantec-Italy). The limit of detection was set at 7.0 pg/mL and 0.14 ng/mL respectively. The Inhibin B normal post-pubertal range for our laboratory is 80–380 pg/mL.

### Semen analysis

The semen samples were collected by means of masturbation directly into a sterile plastic container after a 2–7 day period of sexual abstinence, according to WHO 2010. This criteria was strictly observed because of the possible influence on the study outcome (18). They were examined in accordance to the 2010 World Health Organization criteria (13) and the following variables were taken into consideration: sperm concentration (n × 10^6^/ml), total sperm number (n × 10^6^/ejac.), total motility (%) and morphology (% abnormal forms).

One aliquot of each of the raw semen samples was treated for sperm DNA integrity.

### DNA fragmentation

The DFI was determined by fluorescence microscopy using terminal deoxynucleotidyl Transferase-mediated d-UTP Nick-end Labeling (TUNEL) assay (*In situ* Cell Death Detection Kit, Fluorescein; Roche, Basel, Switzerland) following the procedure described by Gandini et al. (4). One aliquot of each of the raw semen samples was washed twice with NaCl 0.9% solution, the pellets resuspended in PBS containing 10% glycerol to a final sperm concentration of 10 × 10^6^/mL and transferred to Eppendorf snap-cap tubes. The tubes were stored at −80°C. After thawing samples were then cytocentrifuged for 5 min at 200 g and then incubated with a solution of 0.1% Triton X-100 (Sigma, St Louis, MO, USA; T-8787) and 0.1% Sodium Citrate (Sigma, C-8532) for 2 min on ice. After two washing cycles, the slides were air dried and 30 μl of TUNEL mixture (terminal deoxynucleotidyl Transferase and fluorescein-dUTP) were added to each sample. Slides were covered with 22x22 mm coverslips. Samples were incubated 60 min in a moist chamber in the dark, washed three times with PBS and then analyzed under fluorescence microscope (Leica DMR; Leica, Wetzlar, Germany), counting at least 500 cells. The analyses were centrally performed at the Core Laboratory of Seminology (Department of Experimental Medicine University of Rome “La Sapienza”, Italy).

### Statistical analysis

The sample size of the study was calculated by considering a difference between the baseline and the final DFI equal or greater than two percentage points, with a Standard Deviation (SD) of 6.5. The calculation was performed assuming a normal distribution of DFI, using a two sided *t*-test, with a statistical power of 90% and α-error = 0.05. The sample size calculation indicated that 113 would be a suitable number of patients to enrol in the study.

The population normality was verified by using the Kolmogorov-Smirnoff and Shapiro-Wilk tests. Differences between the DFI, before and after treatment, have been verified by the *t*-test for paired samples or the Wilcoxon signed-rank test, depending on the distribution of the DFI changes. The statistical analysis of the hormonal parameters was performed using both the *t*-test for paired samples and the Wilcoxon signed-rank test. The efficacy endpoints were considered as present or absent for the main analysis. For the definition of efficacy, the null hypothesis was that no change in the DFI occurred after treatment, therefore, this statistically significant result was expected if a difference of at least two percentage points between baseline and Visit 2 was detected. Univariate analysis was performed to verify the null hypothesis. In order to compare the results for the DFI pre- and post-treatment parameters, the relative efficacy for the DFI parameter X as (X_t_ – X_0_)/X_0_, where X_0_ is the pre-treatment value and X_t_ is the value of the DFI parameter post treatment were subsequently calculated. The linear regression analysis was performed considering the DFI as a dependent variable.

Statistical analysis was performed with the SPSS® software (version 17 and version 20). Confidence intervals at 95% were computed and statistical tests were performed at a 5% significance level.

## Results

**Figure 2:**
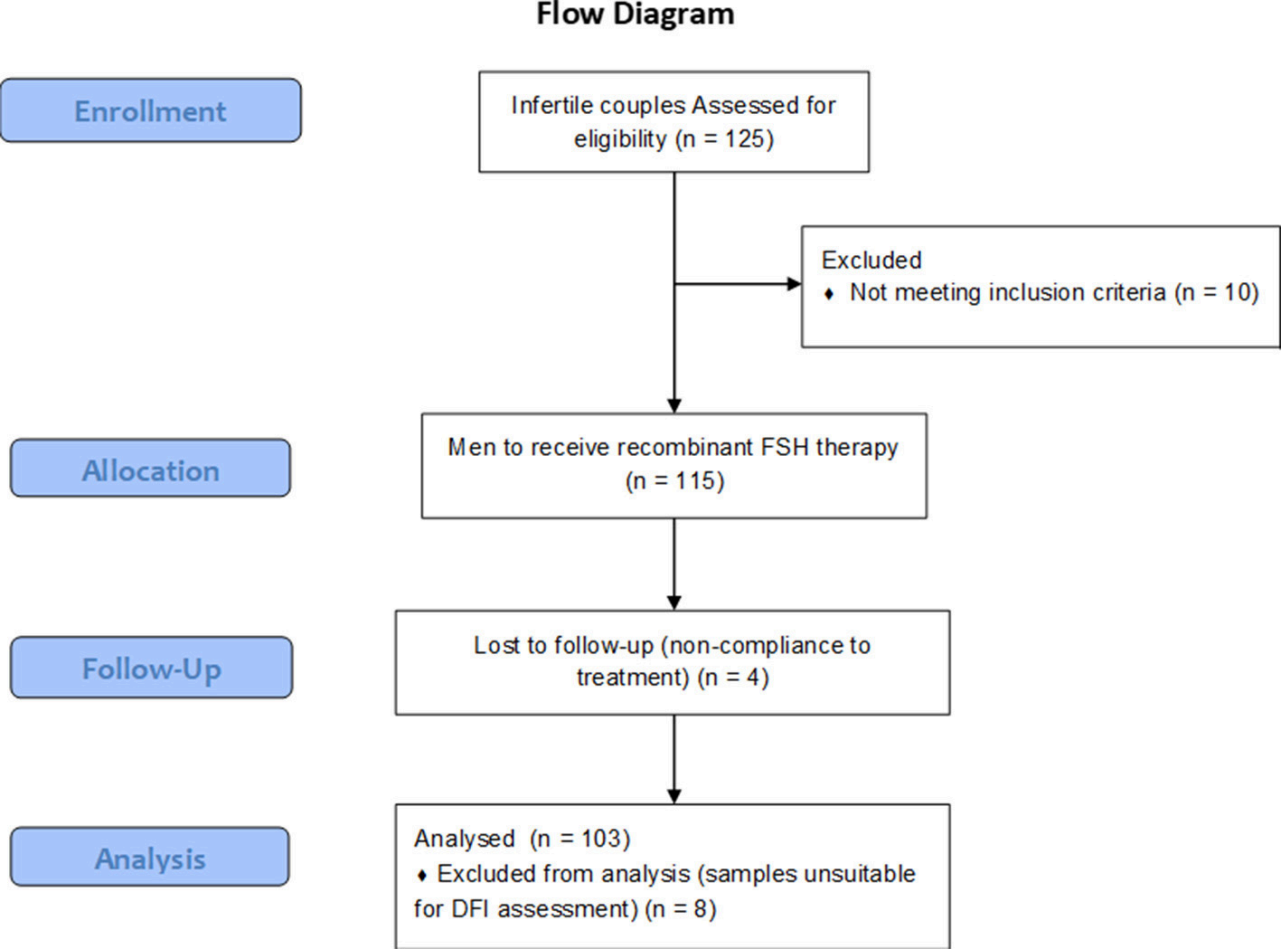
Study flowchart.

One hundred and twenty-five eligible couples were screened and 115 men were enrolled in accordance to the inclusion and the exclusion criteria. During the treatment phase, four drop-outs were recorded and a total of 111 patients completed the study (Figure 2). The power of the analysis performed, was 89.7%. Six adverse events were recorded during the study, although none of these was life threating and in all cases it was a transitory event. The average age of the participants was 36.09 ± 4.67 years old (range 24–46 years). The sperm DNA fragmentation of 8 patients was not assessable due to the unsuitability of the collected sample. These patients were excluded from the statistical analysis, and 103 patients were evaluated for DFI, semen parameters and hormone parameters, pre- and post-treatment. Table 1S reports age, smoking behavior and anthropometric characteristics of the evaluated patients.

### Primary endpoint: DFI change from the baseline before and after treatment

A change from the baseline of the DFI was investigated for the overall population, as well as for the sub-groups obtained through stratification of the whole sample on the basis of the DFI level, age and smoking behavior. The DFI average for all patients at baseline was 18.41 ± 7.86%. After treatment, the DFI showed a significant decrease of 35.4% (15.11 ± 5.97%) (*p* < 0.001) (Table 1). In particular, 69 patients (67%) showed a significant DFI improvement compared to the baseline (*p* < 0.001), whereas, 34 patients (33%) showed significant DFI worsening after treatment (*p* < 0.001).

**Table 1:** DFI, semen and hormonal parameters of 103 patients' baseline and post-treatment (mean, SD).

	**Baseline (visit 0)**	**Post-treatment (visit 2)**	***p*-value**
DFI (%)	18.41 ± 7.86%	15.11 ± 5.97	<**0.001**
Seminal volume (mL)	4.31 ± 9.05	3.15 ± 1.31	0.181
Sperm concentration (×10^6^/ml)	60.03 ± 63.89	101.91 ± 122.512	**0.001**
Total sperm number (×10^6^/ejac)	223.38 ± 284.60	369.28 ± 520.987	<**0.001**
Sperm motility (%)	20.31 ± 7.32	40.37 ± 17.09	<**0.001**
Abnormal forms (%)	83.83 ± 14.27	78.43 ± 15.15	**0.005**
FSH (IU/L)	3.34 ± 1.56	5.74 ± 2.01	<**0.001**
LH (IU/L)	3.09 ± 1.40	2.84 ± 1.30	**0.032**
Testosterone (nmol/L)	5.38 ± 6.05	4.99 ± 2.25	0.521
SHBG (nmol/L)	32.02 ± 12.54	32.72 ± 12.32	0.668
AMH (IU/L)	4.01 ± 2.13	5.32 ± 4.05	<**0.001**
Inhibin B (pg/mL)	160.64 ± 66.75	177.94 ± 77.95	<**0.001**

*Statistically significant differences are in bold*.

**Figure 3:**
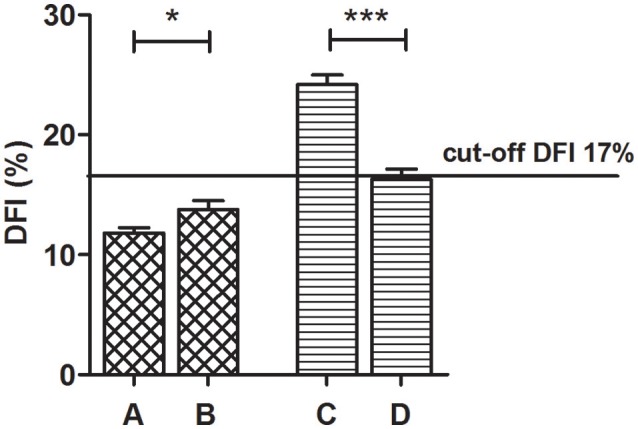
Comparison of the baseline and the post-treatment DFI mean values, by dividing patients in accordance to the median DFI baseline value of 17% (DFI < 17% – 48 patients and DFI ≥ 17% – 55 patients) (Wilcoxon signed-rank test). **(A)** Baseline DFI < 17%, **(B)** Post treatment DFI < 17%, **(C)** Baseline DFI ≥ 17%, **(D)** Post treatment DFI ≥ 17%. ^*^P < 0.05 ^***^P < 0.001.

Age, smoking behavior, hormone levels and semen parameters of the sub-groups obtained through stratification of the whole sample on the basis of the DFI improvement, are shown in Table 2 Sa,b,c. Considering the entire group of patients studied, we observed that the median value of the DFI at baseline was 17%. Thus, we divided patients into two sub-groups on the basis of the median (17%). Forty-eight patients (47%) showed a DFI baseline below 17% and fifty-five patients (53%) showed a DFI higher than 17%. The DFI significantly improved of 32.6% compared to baseline after treatment in the group of patients with a baseline DFI ≥ 17% (24.18 vs. 16.29% respectively; *p* < 0.001). On the contrary, in the sub-group of patients with a baseline DFI <17%, a significant increase, from baseline to post-treatment of the DFI (11.80 vs. 13.76%; *p* < 0.05) with relative difference of 16.6%, was observed (Figure 3, Table 3Sa,b,c).

**Figure 4:**
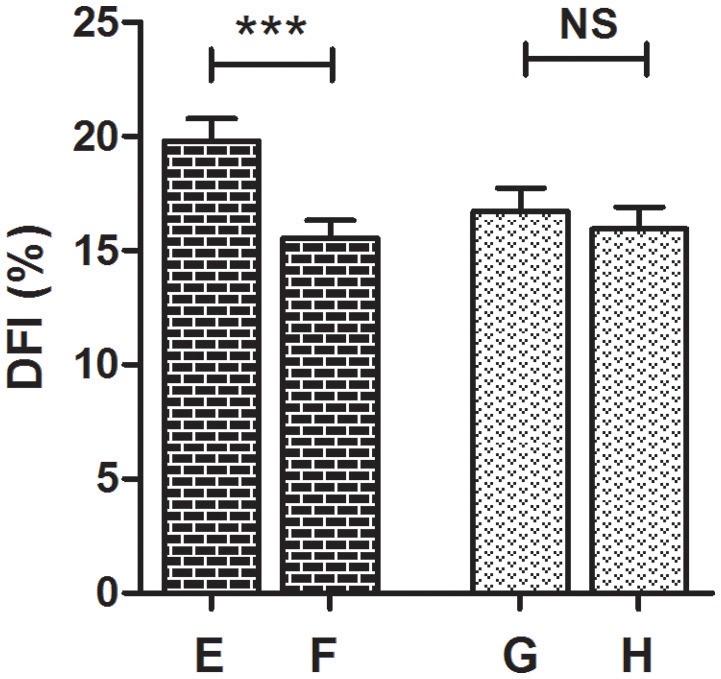
Comparison of the baseline and the post-treatment DFI mean values in smokers (34 patients) and non-smokers (69 patients) (Wilcoxon signed-rank test). **(A)** Baseline, non-smokers, **(B)** post treatment, non-smokers, **(C)** baseline, smokers, **(D)** post treatment, smokers. ^***^P < 0.001, NS, not significant.

Considering the risk factors of the DNA fragmentation, the entire group of patients was divided into two separate groups: smokers (34 patients, 33%) and non-smokers (69 patients; 67%). The DFI improved in 72% (50/69) of the non-smokers and in 56% (19/34) of the smokers. This result suggests that the FSH treatment improves the DFI especially in non-smoking patients (*p* < 0.001), rather than in the smoking patients (*p* = 0.309) (Figure 4).

No significant differences of the DFI improvement were recorded when dividing patients in relation to their age group (data not shown).

Finally, patients were divided in quartiles according to the FSH basal levels distribution (cut-offs: 2.16, 3.13, and 4.27 IU/L). The DFI improvement after treatment was significant in the group of patients with FSH between 2.16 and 3.13 IU/L (17.59 ± 8.08% vs. 13.66 ± 5.80%, *p* = 0.038) and between 3.13 and 4.27 IU/L (19.71 ± 10.30% vs. 15.59 ± 6.91%, *p* = 0.038). This result suggests that the FSH efficacy is less evident when the FSH basal levels were below the first quartile detected in our patients or, on the other hand, higher than the highest quartile. However, since we did not investigate any FSH receptor polymorphism, results should be interpreted accordingly.

### Secondary efficacy endpoints: change from the baseline of the seminal parameters

A significant improvement (*p* < 0.001) of sperm concentration and total sperm number (in 72% of the patients), total sperm motility (97%) and morphology (72%) was recorded after treatment. Semen volume did not change after the FSH treatment (*p* = 0.737) (Table 1).

A linear regression analysis was performed using the DFI as a dependent variable and all seminal parameters as independent variables. A significant inverse relationship was found, both at baseline and after treatment, between the DFI and both sperm concentrations (*p* = 0.041 and *p* = 0.001, respectively) and abnormal forms (*p* < 0.001 and *p* = 0.033, respectively). No differences were seen between the DFI and other seminal parameters.

**Figure 5:**
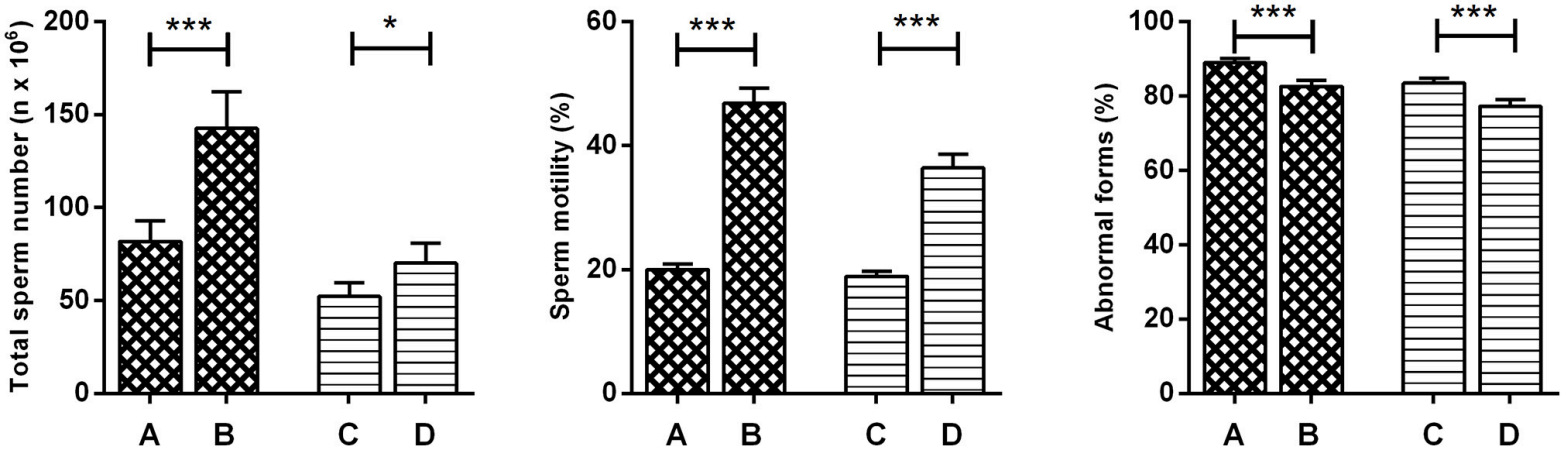
Variation in mean semen parameters of 103 patients, baseline and post-treatment, divided in the sub-group with DFI < 17% (48 patients) and DFI ≥ 17% (55 patients) (Wilcoxon signed-rank test). **(A)** Baseline DFI < 17%, **(B)** post treatment DFI < 17%, **(C)** baseline DFI ≥ 17%, **(D)** post treatment DFI ≥ 17%. ^*^P < 0.05 ^***^P < 0.001.

For the purpose of analysis, patients were divided into sub-groups on the basis of the DFI median (17%). This cut-off was an arbitrary DFI value calculated in accordance with the DFI distribution in the enrolled population. The comparative statistical analysis between the DFI < 17% sub-group and the DFI ≥ 17% sub-group, at baseline, showed a significantly higher total sperm number, and abnormal forms in the DFI < 17% sub-group than in the DFI ≥ 17% sub-group. There was no significant difference found for the sperm motility between the two groups. We observed improvement in all sperm parameters, after treatment, in both subgroups (Figure 5, Table 3S).

### Change from the baseline of the hormonal parameters

The FSH, the Inhibin B and the AMH serum levels significantly increased (*p* < 0.001) after treatment whereas, the LH significantly decreased (*p* = 0.032), even though these values remained within normal range. No significant differences were observed in either the testosterone (*p* = 0.855) or the SHBG average scores (*p* = 0.651).

In the risk analysis of the sub-groups, smokers appeared to be less responsive to treatment, showing weaker improvements in the AMH and the Inhibin B levels when compared to the non-smoker sub-groups.

### Safety

Overall, 6 Adverse Events (AEs) were registered by clinical evaluation during the study. Five patients (4.3%) experienced at least one AE. In five cases, a transient increase of liver enzymes was recorded and in one other case, an alopecia onset was reported. No serious AEs occurred and none of the experienced AEs led to a discontinuation of the study. No severe AEs were experienced by any of the enrolled patients. The AEs were mild and transient and all of them were considered unrelated to the study treatment.

## Discussion

The study demonstrates that recombinant FSH administration, every other day for 3 months, is safe and effective to induce a significant reduction in sperm DNA damage in infertile men. This improvement is significantly higher when the baseline DFI is higher than 17% and the FSH serum levels are between 2.16 and 4.27 IU/L.

The DFI is a well-known marker of sperm quality, and in literature it has been proposed that DFI higher than 30% measured with SCSA is to be considered pathological (19). However, a precise cut-off value may be influenced by different methods used for DNA damage evaluation and to date it is still object of debate. We chose the 17% value, the median of baseline sperm DFI value, as an arbitrary threshold and should not be considered a clinical cut-off value. Here we found that the FSH administration improves the DFI levels of more than 30% compared to baseline. In fact, our study provides a demonstration of the beneficial effects of the FSH administration on spermatogenesis and sperm quality in particular sperm DNA fragmentation but no data on pregnancy outcome was considered. In this setting, the DFI remains a surrogate marker of semen quality and male fertility. The study was designed to evaluate the male partner in infertile couples, thus we are not able to conclude whether this increase is enough to improve the pregnancy rate or the embryo quality.

It is well known that the FSH plays a pivotal role both in spermatogenesis and in sperm DNA maturation. Moreover, the FSH is a key regulator of testis function, required for the establishment of full postnatal development of Sertoli and germ cells and for the maintenance of spermatogenesis in adult men (15). FSH plays a synergistic action with testosterone supporting germ cell survival. For this reason it is plausible that sperm DNA integrity is also positively affected by this hormone (15). Considering this background, nowadays, the recombinant FSH treatment is routinely and empirically used in clinical practice to improve sperm quality and it represents the only available therapy in male idiopathic infertility (10). However, there is no clear evidence of the real beneficial effects of the FSH administration on routine seminal parameters (11). Here, we found that the FSH treatment improves total sperm number, total motility and morphology, suggesting a possible, measurable effect on spermatogenesis. The DFI and the sperm quality are strictly related (especially for the concentration and the morphology aspects), suggesting that the evaluation of these parameters may help the clinicians in the male infertility management.

The DFI seems to be a good marker for sperm quality, even after considering the difficulties and the inter- intra-assay limits (20). Sperm DNA damage could be due to several intrinsic and extrinsic factors. Endogenous damage can be related to protamine deficiency, which leads to the difficulty in the DNA compaction (21). Other involved factors could be age, apoptosis interruption and the increase of oxidative stress (19, 22). On the contrary, known exogenous causes of DNA damage are heat, chemotherapeutic agents, radiations, gonadotoxic agents, cigarette smoking, inflammation of the genital tract, varicocele, and hormone imbalance (23–26). In fact, DNA damage has been associated with altered FSH and LH levels (27). Our findings suggest that infertile men benefit from the FSH administration, in particular, in those cases with elevated DFI baseline levels (DFI > 17%). This subgroup seems to be the optimal target for the treatment cycle with FSH for 3 months. The FSH administration shows a significant effect that is higher in this subgroups, suggesting that these men could better respond to the treatment.

Exogenous agents could influence the sperm DNA integrity and smoke represents an important influencing factor. Here, we excluded from the analysis, heavy smokers (more than 20 cigarettes per day), and we demonstrated an unexpectedly higher mean baseline DFI among the non-smokers, rather than the smokers in our study. However, non-smoking patients (particularly in the younger individuals) are more responsive to the FSH treatment, showing a greater reduction in the DFI. Consistently, non-smokers with high baseline DFI were the ones displaying the best response to treatment concerning the improvements in the sperm DNA damage. These results confirm the role of exogenous agents and oxidative stress as a risk factor for the reduction in male fertility, as well as the predictive role of life-style habits on the response of the FSH treatment. In particular, this finding introduces new variables to be considered when choosing which patient should or should not be treated. However, further properly designed trials should be taken into consideration in order to evaluate the precise action of the FSH on testis and seminal glands and to find out in which spermatogenetic step the FSH acts in.

Although the vast majority of our patients show a DFI improvement after FSH administration, 33% of our patients do not benefit from the therapy. Sub-analyses showed that non-responders are typically men with FSH basal levels higher than 4.27 IU/L or basal DFI lower than 17%.

This DFI value is arbitrary and may not be generalized. In fact, for TUNEL assay used in this study no clear threshold value exist to define normal or abnormal sperm quality. Many threshold values were suggested, ranging from 4 to 35%, and such wide variability is explained by the absence of standardized laboratory protocols for TUNEL assay (28). For this reason in our study sperm DNA integrity analyses were performed in a centralized Laboratory.

Further specific investigations should be carried out in order to better clarify the reason for the lack of benefit observed among these subjects. We could speculate that this finding might be due to the co-occurrence of other undiagnosed alterations, which may affect the spermatogenesis and the sperm DNA condensation. Furthermore, we cannot exclude a correlation between the response to the FSH treatment and the presence of the FSH receptor and the FSH-beta chain polymorphisms. In fact, the occurrence of the FSH receptor polymorphisms may lead either to an impairment in cell response to therapy (14, 29) or to variable spermatogenic activity (15, 30). Only one clinical trial has been reported in the literature showing that these polymorphisms are able to influence the response to the FSH administration (12). Thus, the role of genetic asset of gonadotropins and their receptors should be consider in a fully study of male infertility and to better address the treatment. These genetic parameters could be useful in the near future to recognize responders before the treatment.

The study treatment is associated with a general enhancement of the gonadal function, as described by the improvement of both hormonal (AMH and Inhibin B) and seminal parameters, although no changes are found in testosterone and SHBG serum levels. The AMH increase obtained after FSH administration confirms the action of this gonadotropin on Sertoli cells. Thus, we could speculate in favor to the use of this parameter in the next future to evaluate the efficacy of FSH treatment in male infertility. In particular, the increase observed in the AMH and in the Inhibin B levels seems to be further highlighted among non-smokers, which should confirm the negative impact of oxidative stress on gonadal functions. However, given the small sample size of each sub-group, these results are merely indicative and should be supported by further investigations in larger populations.

Recent studies confirmed that the sperm DNA breaks is a process of apoptosis triggered by several testicular conditions and by oxidative stress during the transit in the male genital tract (31). The clarification of the exact mechanisms leading to the DNA breaks may help to better design studies aimed at evaluating the effects of specific drugs for male infertility, duration and regimen. Finally, although this was a Phase II study, by recruiting and treating a limited number of patients, preliminary conclusions concerning the efficacy and safety of the recombinant FSH 150IU (Gonal-f® PEN 900 IU) in the treatment of the male partner of infertile couples can be drawn.

This study shows several limitations, due to the relative low number of patients treated and the lack of a placebo group and control group. The latter remains the main challenge in the setting of both female and male infertility treatment. In this setting several emotional concerns should be considered and a high time and cost required during the study. Thus, so far, it is impossible to design a classical double-blind, placebo controlled, clinical trial, although it should be the only scientific method to better address the treatment response. Also, FSHR polymorphisms that might influence *in vivo* response to FSH treatment (such as SNP rs6166), were not investigated. A stratification for these parameters might have provided informative results.

Moreover, the study was carried out according to regulations provided by the AIFA note number 74. However, no clear demonstration on the correct dose and duration of the treatment is available at this time. Taking into consideration the duration of spermatogenesis, further studies should be designed concerning the different therapeutic approaches to confirm the beneficial role of FSH in male infertility.

## Conclusion

This study provided encouraging, effective results and an adequate level of confidence concerning safety, suggesting to proceed to Phase III *ad hoc* studies in larger populations, aimed at exploring the FSH potential efficacy in infertile men. These trials may be useful to evaluate if the male treatment could improve major outcomes, such as embryo quality and pregnancy rate during assisted reproductive techniques.

## Availability of data and material

The datasets used and/or analyzed during the current study are available from the corresponding author on reasonable request.

## Author contributions

The concept and design of the study: NC, FL, SL, ML, VDL, EP, and RD. The analysis and interpretation of data: FP, DP, PP, GR, ELV, FC, and RP. The drafting of the article: NC, FL, DP, SL, and ML. The revision of the paper: All authors. All authors read and approved the final manuscript.

### Conflict of interest statement

ML and SL are employed by Merck-Serono; RD has received Institutional and Research grants from MSD and Merck-Serono; RP has received Institutional and Research grants from Merck-Serono and IBSA; EP has received grants and honoraria, and consults for MSD, Merck-Serono, Ferring, and IBSA Institut Biochimique SA. This trial was sponsored by Merck Serono S.p.A., an affiliate of Merck KGaA, (Darmstadt, Germany). The remaining authors declare that the research was conducted in the absence of any commercial or financial relationships that could be construed as a potential conflict of interest. The handling Editor declared a shared affiliation, though no other collaboration, with the authors NC, FC, RP, EV at time of review.

## Abbreviations

AE: Adverse Event
AIFA: Italian Medicines Agency
AMH: Anti-mullerian hormone
DFI: DNA Fragmentation Index
FSH: Follicle-Stimulating Hormone
hCG: Chorionic Gonadotropin
HH: Hypogonadotropic Hypogonadism
ICSI: Intracytoplasmic Sperm Injection
LH: Luteinizing Hormone
SHBG: Sex Hormone Binding Globulin
TUNEL Assay: Terminal Deoxynucleotidyl Transferase-Mediated d-UTP Nick-end Labeling Assay.
