# Supplementary material for: Recombinant FSH Improves Sperm DNA Damage in Male Infertility: A Phase II Clinical Trial
Source: Front Endocrinol (Lausanne). 2018 Jul 10;9:383. doi: 10.3389/fendo.2018.00383 (PMC6048873; doi:10.3389/fendo.2018.00383)
Supplement: Supplementary file 1 [file Table_1.DOCX]

| **Overall Study Population** | | | |
| --- | --- | --- | --- |
|  | **Mean** | **SD** | **Median** |
| **Age (Years)** | 36,09 | 4,67 | 36 |
| **Weight** | 82,10 | 11,56 | 80 |
| **Height** | 177,09 | 6,15 | 177 |

**Table 1 S** – Anthropometric characteristics of the overall study population
