# Supplementary material for: Recombinant FSH Improves Sperm DNA Damage in Male Infertility: A Phase II Clinical Trial
Source: Front Endocrinol (Lausanne). 2018 Jul 10;9:383. doi: 10.3389/fendo.2018.00383 (PMC6048873; doi:10.3389/fendo.2018.00383)
Supplement: Supplementary file 2 [file Table_2.DOCX]

|  | Age (years) | Smokers (%) | Volume (ml) | Sperm concentration (x 10^6^) | Total Motility (%) | Abnormal forms (%) | FSH (IU/l) | LH  (IU/l) | TE (ng/ml) | SHBG (nmol/l) | INHB (pg/ml) | DFI (%) |
| --- | --- | --- | --- | --- | --- | --- | --- | --- | --- | --- | --- | --- |
| Pre-Therapy  (69 pts) | 35.7±4.7  (36) | 19/69 (27.5%) | 3.0±1.3  (3) | 57.8±64.6  (29.4) | 18.6±6.4  (20) | 85.9±9.7  (89) | 3.4±1.7  (3.2) | 3.0±1.4  (2.9) | 4.8±1.6  (4.6) | 33.4±11.8  (34) | 157.3±63.3  (142) | 21.3±7.5  (20) |
| Post-therapy  (69 pts) | // | // | 3.0±1.3  (3) | 87.1±113.6  (52) | 39.4±18.8  (40) | 80.4±12.7  (86) | 5.8±2.1  (5.8) | 2.9±1.4  (2.7) | 4.8±1.6  (4.5) | 32.6±12.0  (31.6) | 176.3±76.5  (159.5) | 13.6±5.6  (12.2) |
| P value^a^ | N/A | N/A | ns | 0.001 | <0.001 | <0.001 | <0.001 | ns | ns | ns | <0.001 | <0.001 |

**Table 2 S a** – Patients responsive to FSH treatment (with DFI improvement): means ± SD and medians (in brackets) of age, smokers, sperm parameters and hormone levels pre- and post-therapy.

**Table 2 S b** – Patients non-responsive to FSH treatment (without DFI improvement): means ± SD and medians (in brackets) of age, smokers, sperm parameters and hormone levels pre- and post-therapy.

|  | Age (years) | Smokers (%) | Volume (ml) | Sperm concentration (x 10^6^) | Total Motility (%) | Abnormal forms (%) | FSH (IU/l) | LH  (IU/l) | TE (ng/ml) | SHBG (nmol/l) | INHB (pg/ml) | DFI (%) |
| --- | --- | --- | --- | --- | --- | --- | --- | --- | --- | --- | --- | --- |
| Pre-Therapy  (34 pts) | 36.7±4.7  (37) | 15/34 (44.1%) | 3.4±1.4  (3) | 82.7±69.7  (68) | 21.2±5.5  (23.5) | 86.4±8.8  (90) | 3.3±1.2  (3.1) | 3.2±1.4  (3.2) | 4.9±1.4  (4.8) | 33.2±11.3  (33.6) | 164.7±69.8  (150.5) | 13.1±5.7  (12.2) |
| Post-therapy  (34 pts) | // | // | 3.6±1.4  (3.5) | 138.0±115.4  (106) | 45.4±12.7  (44) | 78.5±13.5  (85) | 5.6±2.0  (5.7) | 2.8±1.3  (2.8) | 4.9±1.2  (4.7) | 33.3±11.6  (33.9) | 182.7±84.1  (157.5) | 18.2±5.6  (16.9) |
| p value^a^ | N/A | N/A | 0.31 | <0.001 | <0.001 | <0.001 | <0.001 | ns | ns | ns | <0.001 | <0.001 |

**Table 2 S c** – Comparison of pre-therapy sperm parameters and hormone levels between patients responsive and non-responsive to FSH treatment: means ± SD and medians (in brackets).

|  | Age (years) | Smokers (%) | Volume (ml) | Sperm concentration (x 10^6^) | Total Motility (%) | Abnormal forms (%) | FSH (IU/l) | LH  (IU/l) | TE (ng/ml) | SHBG (nmol/l) | INHB (pg/ml) | DFI (%) |
| --- | --- | --- | --- | --- | --- | --- | --- | --- | --- | --- | --- | --- |
| Responsive  (69 pts) | 35.7±4.7  (36) | 19/69 (27.5%) | 3.0±1.3  (3) | 57.8±64.6  (29.4) | 18.6±6.4  (20) | 85.9±9.7  (89) | 3.4±1.7  (3.2) | 3.0±1.4  (2.9) | 4.8±1.6  (4.6) | 33.4±11.8  (34) | 157.3±63.3  (142) | 21.3±7.5  (20) |
| Non-responsive  (34 pts) | 36.7±4.7  (37) | 15/34 (44.1%) | 3.4±1.4  (3) | 82.7±69.7  (68) | 21.2±5.5  (23.5) | 86.4±8.8  (90) | 3.3±1.2  (3.1) | 3.2±1.4  (3.2) | 4.9±1.4  (4.8) | 33.2±11.3  (33.6) | 164.7±69.8  (150.5) | 13.1±5.7  (12.2) |
| p value^b^ | ns | ns^c^ | ns | p = 0.03 | p = 0.05 | ns | ns | ns | ns | ns | ns | p < 0.001 |

^a^ Wilcoxon signed rank test (pre- vs post-therapy)

^b^ Mann Whitney U test (Basal DFI improved vs Basal DFI not improved) unless specified

^c^ Fisher Exact test (p = 0.11)
