# Supplementary material for: Recombinant FSH Improves Sperm DNA Damage in Male Infertility: A Phase II Clinical Trial
Source: Front Endocrinol (Lausanne). 2018 Jul 10;9:383. doi: 10.3389/fendo.2018.00383 (PMC6048873; doi:10.3389/fendo.2018.00383)
Supplement: Supplementary file 3 [file Table_3.DOCX]

**Table S 3a** – Baseline means ± SD and medians (in brackets) of age, smokers, sperm parameters and hormone levels of with DFI < 17% (48 patients) and DFI ≥ 17% (55 patients) sub-groups.

|  | Age (years) | Smokers (%) | Volume (ml) | Sperm concentration (x 10^6^) | Total Motility (%) | Abnormal forms (%) | FSH (IU/l) | LH  (IU/l) | PRL | TE (ng/ml) | SHBG (nmol/l) | INHB (pg/ml) | DFI (%) |
| --- | --- | --- | --- | --- | --- | --- | --- | --- | --- | --- | --- | --- | --- |
| DFI < 17% Pre therapy  (48 pts) | 36.4±4.7  (37) | 22/48 (45.8%) | 3.0±1.3  (3) | 81.8±76.5  (51.8) | 20±6.2  (22) | 89.0±8.3  (92) | 3.5±1.7  (3.2) | 3.4±1.4  (3.5) | 8.3±3.8  (8) | 4.7±1.3  (4.7) | 32.0±11.2  (33.3) | 152.3±58.1  (144) | 11.8±3.2  (12.5) |
| DFI ≥ 17% Pre therapy  (55 pts) | 35.7±4.8  (36) | 12/55 (21.8%) | 3.2±1.4  (3) | 52.2±54.5  (27.5) | 18.9±6.3  (20) | 83.5±9.5  (80) | 3.3±1.4  (3.2) | 2.8±1.3  (2.6) | 8.3±3.2  (8) | 5.0±1.7  (4.7) | 34.5±11.8  (34) | 165.8±70.5  (155) | 24.2±5.9  (22.8) |
| P value | 0.258 | 0.012 | 0.412 | 0.031 | 0.579 | 0.003 | 0.794 | 0.003 | 0.726 | 0.404 | 0.361 | 0.332 | <0.001 |

**Table S 3b** – Variation of semen parameters and hormone levels baseline vs post-treatment of the sub-group with DFI < 17% (48 patients): means ± SD and medians (in brackets).

|  | Volume (ml) | Sperm concentration (x 10^6^) | Total Motility (%) | Abnormal forms (%) | FSH (IU/l) | LH  (IU/l) | PRL | TE (ng/ml) | SHBG (nmol/l) | INHB (pg/ml) | DFI (%) |
| --- | --- | --- | --- | --- | --- | --- | --- | --- | --- | --- | --- |
| DFI < 17% Pre therapy  (48 pts) | 3.0±1.3  (3) | 81.8±76.5  (51.8) | 20±6.2  (22) | 89.0±8.3  (92) | 3.5±1.7  (3.2) | 3.4±1.4  (3.5) | 8.3±3.8  (8) | 4.7±1.3  (4.7) | 32.0±11.2  (33.3) | 152.3±58.1  (144) | 11.8±3.2  (12.5) |
| DFI < 17% Post therapy  (48 pts) | 3.2±1.4  (3) | 142.6±138.0  (75.8) | 46.8±16.8  (46) | 82.7±11.4  (88) | 6.1±2.1  (5.9) | 2.9±1.3  (2.7) | 8.2±3.9  (7) | 4.8±1.4  (4.7) | 32.1±11.4  (33.2) | 171.6±70.8  (156.5) | 13.8±5.2  (13.2) |
| P value | 0.154 | <0.001 | <0.001 | <0.001 | <0.001 | 0.006 | 0.325 | 0.431 | 0.845 | <0.001 | 0.026 |

**Table S 3c** – Variation of semen parameters and hormone levels baseline vs post-treatment of the sub-group with DFI ≥ 17% (55 patients): means ± SD and medians (in brackets).

|  | Volume (ml) | Sperm concentration (x 10^6^) | Total Motility (%) | Abnormal forms (%) | FSH (IU/l) | LH  (IU/l) | PRL | TE (ng/ml) | SHBG (nmol/l) | INHB (pg/ml) | DFI (%) |
| --- | --- | --- | --- | --- | --- | --- | --- | --- | --- | --- | --- |
| DFI ≥ 17% Pre therapy  (55 pts) | 3.2±1.4  (3) | 52.2±54.5  (27.5) | 18.9±6.3  (20) | 83.5±9.5  (80) | 3.3±1.4  (3.2) | 2.8±1.3  (2.6) | 8.3±3.2  (8) | 5.0±1.7  (4.7) | 34.5±11.8  (34) | 165.8±70.5  (155) | 24.2±5.9  (22.8) |
| DFI ≥ 17% Post therapy  (55 pts) | 3.1±1.3  (3) | 70.1±80.0  (45) | 36.4±16.1  (35) | 77.3±13.7  (80) | 5.4±2.0  (5.6) | 2.8±1.3  (2.6) | 8.3±3.4  (8) | 4.9±1.5  (4.7) | 33.5±12.3  (31.6) | 184.5±85.4  (164) | 16.3±6.3  (15.1) |
| P value | 0.247 | 0.014 | <0.001 | <0.001 | <0.001 | 0.722 | 0.982 | 0.632 | 0.253 | <0.001 | <0.001 |
